# A data-driven mathematical model of multi-drug resistant Acinetobacter baumannii transmission in an intensive care unit

**DOI:** 10.1038/srep09478

**Published:** 2015-03-25

**Authors:** Xia Wang, Yong Chen, Wei Zhao, Yan Wang, Qing Song, Hui Liu, Jingya Zhao, Xuelin Han, Xiaohua Hu, Hajo Grundmann, Yanni Xiao, Li Han

**Affiliations:** 1 Department of Applied Mathematics, Xi'an Jiaotong University, Xi'an 710049, China; 2 Center for Hospital Infection Control, Chinese PLA Institute for Disease Control and Prevention, 20# Dongda Street, 100071 Beijing, China; 3 Department of Surgical Intensive Care Unit, Chinese PLA General Hospital, 100853 Beijing, China; 4 Department of Medical Microbiology, University Medical Centre Groningen, Groningen, The Netherlands

## Abstract

Major challenges remain when attempting to quantify and evaluate the impacts of contaminated environments and heterogeneity in the cohorting of health care workers (HCWs) on hospital infections. Data on the detection rate of multidrug-resistant *Acinetobacter baumannii* (MRAB) in a Chinese intensive care unit (ICU) were obtained to accurately evaluate the level of environmental contamination and also to simplify existing models. Data-driven mathematical models, including mean-field and pair approximation models, were proposed to examine the comprehensive effect of integrated measures including cohorting, increasing nurse-patient ratios and improvement of environmental sanitation on MRAB infection. Our results indicate that for clean environments and with strict cohorting, increasing the nurse-patient ratio results in an initial increase and then a decline in MRAB colonization. In contrast, in contaminated environments, increasing the nurse-patient ratio may lead to either a consistent increase or an initial increase followed by a decline of MRAB colonization, depending on the level of environmental contamination and the cohorting rate. For developing more effective control strategies, the findings suggest that increasing the cohorting rate and nurse-patient ratio are effective interventions for relatively clean environments, while cleaning the environment more frequently and increasing hand washing rate are suitable measures in contaminated environments.

*Acinetobacter* is a genus of Gram-negative bacteria that live in the soil and water as well as on the skin. Decades ago, researchers discovered that *Acinetobacter baumannii* had acquired resistance to antimicrobial drugs such as most aminoglycosides, first- and second-generation cephalosporins, and ureidopenicillins^1^. Since then, strains of *A. baumannii* have continued to develop resistance to new antibiotics. Although multidrug-resistant *A. baumannii* (MRAB) does not make healthy people sick, it can cause serious infections in wounds, lungs and blood. As such, it is not usually seen in the community as extensively as it is in hospitals. Infections caused by MRAB are difficult to treat because many antibiotics are ineffective against it^2^. Therefore, MRAB has become an important cause of hospital-acquired infection.

Studies have shown that *A. baumannii* strains could be isolated from a hospital bed rail of an infected patient and kept alive for 9 days after their discharge^3^. Wendt et al.^4^ showed that *A. baumannii* strains isolated from dry sources had better survival rates than those isolated from wet sources. *Acinetobacter* spp. have been identified on inanimate hospital objects up to 5 months after transmission. Ventilators, suctioning equipment, mattresses and sinks are some of the more common sites that tend to remain colonized for extended periods. As such, *A. baumannii* strains in the environment are important causes of infection that cannot be ignored. Colonization with *A. baumannii* can result via contact with a human carrier^12^ or a contaminated environment. Some mathematical models have been established to analyze the effect of free-living environmental bacteria^5^,^6^,^7^,^8^,^9^,^10^,^11^ buteffects of the contaminated environment in these models were implicitly investigated by using data on case numbers of hosts since no reliable data on levels of environmental contaminations were available. Thus, it remains challenging to accurately quantify and evaluate the impact of contaminated environments on hospital infection. Therefore, this study was designed to determine how to assess an environment's level of contamination and consequently propose a suitable mathematical model to examine the effect of free-living bacteria in the environment on hospital-acquired infections.

There is some evidence showing that the ratio of HCWs to patients greatly affects epidemic severity^13^,^14^. A number of papers have investigated the effect of HCW-patient ratios on hospital infection by formulating mathematical models that examine cohorting^15^,^16^,^17^, one of the most important measures used to reduce the transmission of bacteria in the hospital setting. The existing models generally assume that the cohorting of HCWs, especially nurses, is homogeneously mixed^15^,^16^,^18^. Little attention has been given to the heterogeneous contacting mechanism amongst cohorted HCWs, uncohorted HCWs, and patients. Determining the number of HCWs that should be staffed on an intensive care unit (ICU) and describing inter-cohort heterogeneity remain challenging, and fall within the scope of this study, the main purpose of which was to propose data-driven mathematical models. These include mean-field and pair-approximation models^19^,^20^,^21^,^22^,^23^,^24^,^25^ to investigate the effect of contaminated environments on hospital-acquired infections to examine the heterogeneous relationship between patients and HCWs and the heterogeneity caused by cohorting. The results of the network modeling work are expected to aid our understanding of how many nurses should be staffed in an ICU and possibly assist in the development of more effective infection control strategies.

## Methods

### Ethics Statement

The study was approved by the Institutional Ethic Committee of Chinese PLA General Hospital, Beijing, China. Written informed consent was obtained from all participants prior to the study. The methods were carried out in accordance with the international ethical guidelines on biomedical research involving human subjects.

### Data

**Figure 1:**
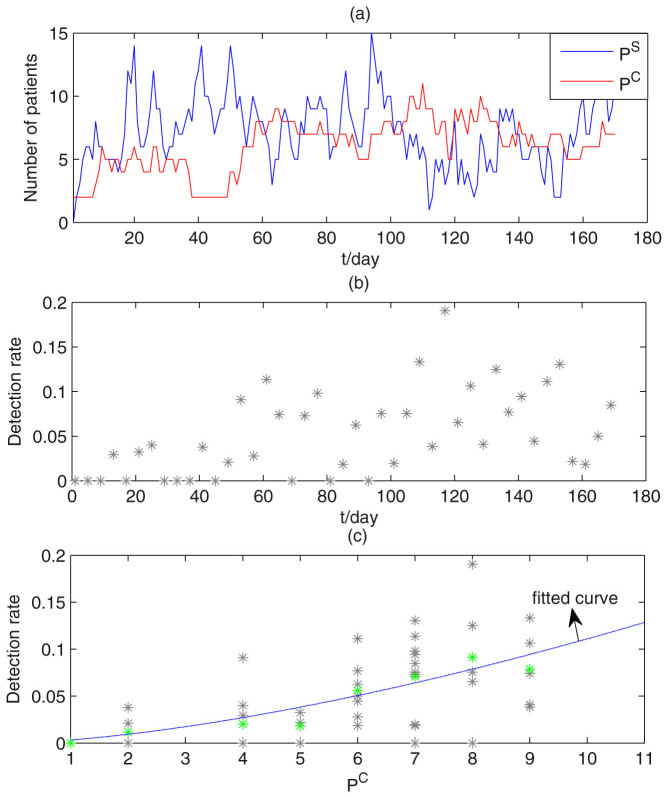
(a) Time series of numbers of colonized and uncolonized Patients per day. Red lines, colonized patients; blue lines, uncolonized patients. (b) Detection rates of MRAB throughout the ward. (c) Correlations between detection rates and number of colonized patients. Gray stars - data; green stars - mean detection rate; the line - fitted curve.

The data were collected in a surgical ICU (SICU) of a 4,000-bed tertiary-care general hospital in Beijing, China. A total of 290 patients was admitted to the SICU (capacity of 20) from July 15, 2008 to December 31, 2008. The data included both the cases of MRAB infection and the detection rate of MRAB in the environment. The data also included admission and discharge times, whether a patient was colonized at the time of admission, and when a susceptible patient became colonized after admission. Of the newly admitted patients, 13 were already colonized with MRAB and 68 acquired MRAB after admission. Fig. 1(a) shows the daily data of MRAB-positive (red curve) and MRAB-negative (blue curve) patients.

The detection rate of MRAB in the environment is defined by the proportion of positive samples, which could represent the concentration of bacteria in the environment, and is shown in Fig. 1(b). Approximately 100 samples of the air, faucets, dispensing stations, and areas surrounding the beds were collected every 4 days. Comparing Figs. 1(a) and (b) suggests that these two data sets have similar trends. Moreover, Pearson's correlation coefficient was calculated to be 0.57 (*p* = 7.24*10^−5^) between the number of colonized patients and the detection rates of bacteria in a ward. We chose a power law function to fit the mean detection rates since it gave a relatively large *R*^2^ coefficient of determination, which is a statistical measure of how well the regression line approximates the real data points (see details in Supporting Information (SI)). Let *W*(*t*) be the detection rate of MRAB in the ICU environment and *P^C^*(*t*) be the number of colonized patients, then we have *W*(*t*) = *a*(*P^C^*(*t*))*^b^*, *a* = 0.0032, *b* = 1.54, and the goodness of fit is shown in Fig. 1(c).

The contact rate was defined as the number of physical contacts^17^ per patient per hour. The contact rates between patients and nurses or doctors were obtained through direct observation for a total of 59 hours over 37 days in August, September, and October 2008. Our data show that contact rates in different periods might be very different, which made it difficult to obtain an accurate contact rate per unit of time. But we could deduce that the contact rate of patients and nurses is approximately 4.6 times greater than that of patients and doctors (see details in SI). This motivated us to model the dynamics of doctors and nurses separately.

### The model equations

The transmission mechanism of MRAB in a ward is via health care workers and patients, which is similar to that of MRSA or VRE^10^,^11^,^15^,^26^. Similarly, a contaminated environment is also an important factor contributing to hospital infections. So, we propose a mathematical model that includes both direct transmission between HCWs and patients and indirect transmission via free-living bacteria in the environment. We assumed that once patients become colonized they remain colonized for the duration of their stay in the ICU since MRAB may result in long-term colonization. Due to the lack of physical contacts among HCWs, we assume that there is no direct transmission between them. Meanwhile, patients contact neither each other nor the common environment due to serious illness^27^.

On the basis of the large differences in contact rates between patients with doctors and of patients with nurses, we model them separately. Let *P^S^* be the number of uncolonized patients and *D^C^*, *H^C^* be the numbers of colonized doctors and nurses, respectively. Suppose the inflow rate of patients to hospital is Λ, and assume that a fraction *φ* of the admitted patients were colonized prior to admission to the ward. Let *d*_1_ (*d*_2_) be the discharge rate of the susceptible patients (colonized patients) and *γ* be the rate of HCW decontamination due to hand washing. The definitions of the parameters are given in Table 1. The model equations are as follows:
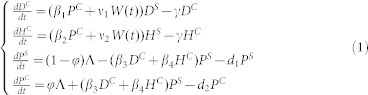
Patients can be colonized via contact with contaminated doctors or nurses (denoted by (*β*_3_*D^C^* + *β*_4_*H^C^*)*P^S^*). Doctors and nurses can be contaminated via contact with colonized patients (denoted by *β*_1_*P^C^D^S^* and *β*_1_*P^C^H^S^*) or contaminated environments (denoted by *v*_1_*WD^S^* or *v*_2_*WH^S^*). Since the detection rate can represent the degree of environmental contamination, the concentration of the bacteria in the environment can be reflected by it. By using the data-determined relationship of number of colonized patients and the detection rates of bacteria in a ward, we then extended the traditional models^7^,^9^,^10^ by including the detection rate of MRAB throughout cases of colonized patients rather than the density of the free-living bacteria. Therefore, our data-driven model has the potential to examine the effect of contaminated environments.

To study the heterogeneous transmission dynamics of MRAB among HCWs and patients, we extended the mean-field model (1) to be a pair approximation model^22^,^23^,^24^,^25^. We consider the spread of MRAB through a network with *N* nodes representing the numbers of doctors, nurses, patients, and empty beds. Nodes that represent doctors, nurses and patients can be contaminated/colonized or susceptible. The state of a node is changed to an empty bed when a patient is discharged from the ward. As such, there are seven types of nodes [*D^S^*], [*D^C^*], [*H^S^*], [*H^C^*], [*P^S^*], [*P^C^*] and [*ϕ*]. Due to symmetries (*AB* = *BA*), there are also twelve distinct types of pairs ([*D^C^P^S^*], [*D^S^P^S^*], [*D^C^P^C^*], [*D^S^P^C^*], [*P^C^H^S^*], [*P^S^H^S^*], [*P^C^H^C^*], [*P^S^H^C^*], [*D^S^ϕ*], [*D^C^ϕ*], [*H^S^ϕ*] and [*H^C^ϕ*]). The average number of neighbors of doctors, nurses and patients are denoted by *Q_D_*, *Q_H_* and *Q_P_* separately, where 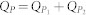 with 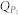 representing the average number of doctors connected to a patient and 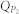 the average number of nurses connected to a patient. Here, it should be noted that both doctors and nurses can contact patients, while connection between them is impossible. Thus, those nodes and pairs satisfy certain equalities given in the SI. Therefore, only the pairs [*D^C^P^C^*] and [*H^C^P^C^*] need to be considered in our model. It is worth mentioning that contacts between HCWs and the contaminated environment are modeled homogeneously due to similarities among contaminated environments for each HCW. The pair-approximation model is as follows:
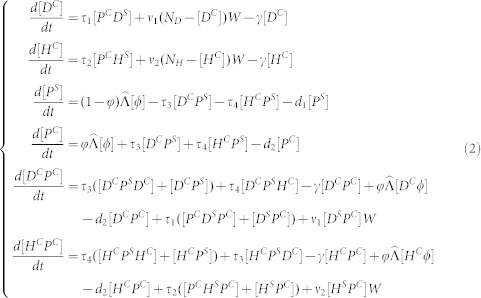
where 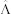 is the admission rate and *τ_i_* (*i* = 1,2,3,4) is the transmission rate on a contact network. According to the work of Rand^24^ and Keeling^22^, we have
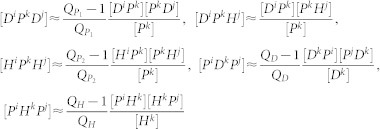
where *i*, *j*, *k* represent *S* or *C*. It is theoretically difficult to analyze this pairwise equation due to its complexity. However, information about the model's behavior can be obtained by numerical simulations.

### Parameter estimation

The admission rate of the patients Λ was 1.72 (/day), while the proportion of patients who were infectious when hospitalized (*φ*) was 0.0448. MRAB-positive and -negative patients had a mean ward stay of 12.9 and 4.2 days, respectively. The outflow rate of MRAB-positive (or -negative) patients *d*_2_ (or *d*_1_) was, therefore, 1/12.9 (/day) (or 1/4.2/day). The total number of beds in the ward (*N_P_*) was 20. The average number of nurses (*N_H_*) and doctors (*N_D_*) managing the ward every day were 15 and 11, respectively. Indirect transmission rates between HCWs and patients *v*_1_ and *v*_2_ (which are difficult to obtain) were assumed to be the same and defined as *v*. The initial patient values were chosen as the first observations in our data set: *P^S^*(0) = 1 and *P^C^*(0) = 2. Since HCWs are susceptible when they start their shifts, we assumed that their initial values were *D^C^*(0) = 0 and *H^C^*(0) = 0 as in the paper of Wolkewitz^28^ and these initial data were applied to all simulations in the paper.

The parameter values were estimated in a Bayesian framework using MCMC simulation by the JAGS Gibb's sampler. Uniform (0.001, 0.1), Uniform (0.0005, 0.01) and Uniform (10, 100) prior distributions were used for *β*_1_, *β*_3_ and *v*. The R Statistical System was used for the simulation. The algorithm runs for 80000 iterations with a burn-in of 8000 iterations, with the Geweke convergence diagnostic method was employed to assess the convergence^29^. The mean values, 95% confidence intervals (95% CI) of the three parameters, and the 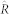 of the Gelman-Rubin statistic are shown in Table 2.

### Stochastic simulation

Stochastic simulation^30^,^31^ is usually considered to realistically represent disease spreading in a network, whereas the pairwise model is an approximation; consequently, stochastic simulation results are considered to be the benchmark. Two kinds of networks: the random network^23^ and the strict cohorting network, were considered to compare and verify the results of the pairwise model. The degrees of both kinds of networks are the same as that of the pairwise model. The detailed configurations of the two networks are given in the SI.

## Results

### Behavior of the proposed models

**Figure 2:**
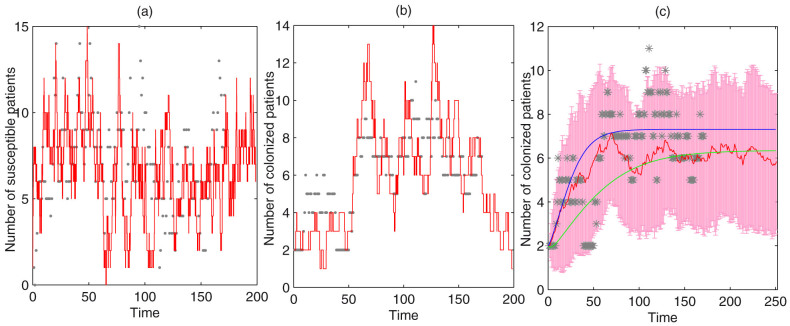
Goodness fit of stochastic simulations on the simulated network with the collected data. (a) ((b)) Number of susceptible patients (colonized patients). The curves in the figure show the simulation results while the dots show the data. (c) Predicted number of colonized patients by the mean-field model (green curve), the pairwise model (blue curve) and the mean and variations (mean ± standard deviation) of stochastic simulations on the random network (red curve). All parameter values are as listed in Table 1.

Fig. 2(a–b) shows the goodness of fit of the stochastic simulations with the data on the susceptible and colonized patients. The curves in this figure represent the stochastic simulation on a random network with the maximum node degree using parameter values listed in Table 1 and the dots represent our acquired data. Fig. 2(c) shows the predicted number of colonized patients for the mean-field model (1) (green curve) and the pairwise model (2) (blue curves). The mean number and its variation (mean ± standard deviation) of colonized patients predicted by 50 stochastic simulations on the random network with the maximum node degree are also shown as the red line in Fig. 2(c). This finding indicates that the number of colonized patients for the mean-field model stabilized at 6.2, slightly less than that (7.2) for the pairwise model. The stochastic simulation results on the network show that the number of colonized patients initially increased and then fluctuated between five and seven after about 100 days. The solutions of the two differential equation models were within the scope of the fluctuation of the stochastic simulation.

### Effect of nurse cohorting and the nurse-patient ratio on MRAB infection

The cohorting measure is represented in our network model by separating and grouping nurses. The cohorting rate *p* is defined as the proportion of nurses who are cohorted. Note that maximum grouping means the number of groups is considered to equal the number of cohorted nurses. As such, the total number of nurses *N_H_* is divided into 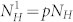 and 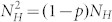. The degree of the cohorted nurses (denoted by 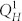) is 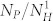, which indicates that they were maximally grouped, and the degree of the other nurses (denoted by 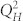) was *N_P_*. The corresponding pairwise model with cohorting of nurses can be written as model (S1) (see the SI).

It follows from Fig. 2(c) that model predictions stabilize at almost fixed values after about 200 days. So, to get a reliable result, in the following we focus on variation in the average number of colonized patients at day 500 with the cohorting rate and/or the ratio of nurses to patients by simulating the pairwise model and the stochastic simulations on the random or strict cohorting networks with a cohorting rate *p*. In each situation, we increased the number of nurses from 1 to 20; hence, the ratio of nurses to patients increased from 0.05 to 1. Note that when we varied *Q_H_*, we scaled the transmission rates on the network *τ_i_* such that transmission rates *β_i_*, *i* = 1,2,3,4 were fixed.

**Figure 3:**
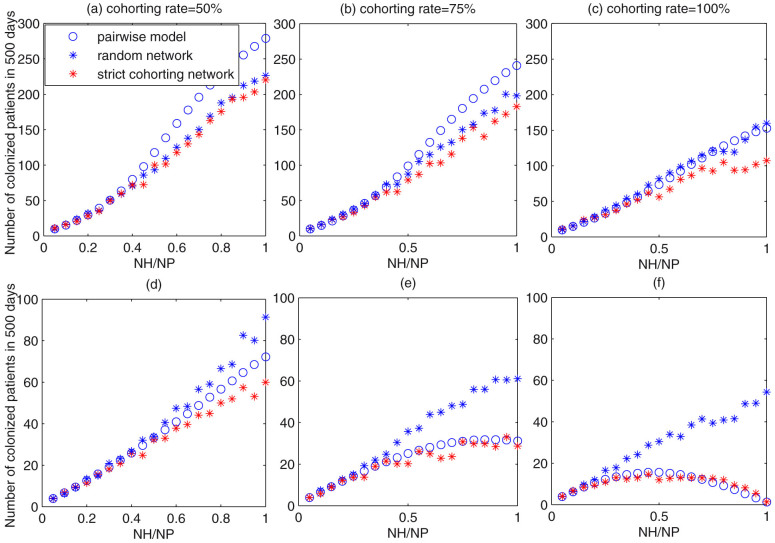
The average number of colonized patients in a period of 500 days versus the nurse-patient ratio when the cohorting rate was 50% ((a), (d)), 75% ((b), (e)), or 100% ((c), (f)). Figures in the first line (a), (b) and (c) show the results when the indirect transmission rate was 48.9, while those in the second line show the results when the indirect transmission rate was 0. Blue circles show simulation results for the pairwise model. Blue (red) stars show simulation results on the random network (the strict cohorting network). All other parameter values are as listed in Table 1.

Figs. 3 (a)–(c) show the predicted numbers of colonized patients versus the nurse-patient ratio when the cohorting rates were 50%, 75% and 100%, respectively. This indicates that increasing the number of nurses leads to increased numbers of colonized patients. Comparison of Figs. 3 (a), (b), and (c) suggests that a greater nurse cohort results in fewer colonized patients. In particular, when the cohorting rate increased by 25% (from 50% to 75%), the maximum value shown in Fig. 3(a) is decreased by less than 50 compared to that in Fig. 3 (b) for the pairwise model. Further, it follows from Fig. 3 (b) and (c) that the value at *N_H_*/*N_p_* = 1 is decreased by almost 100 when the cohorting rate increased by 25% (from 75% to 100%). This finding reveals that the greater the cohorting rate, the more pronounced the effect of increasing the nurse-patient ratio on the number of colonized patients. Therefore, the use of a large cohorting rate has the potential to control disease transmission. It is interesting to note that from Fig. 3 (a)–(c) we obtain that the stochastic simulation results on the random network were almost identical to those on the strict cohorting network for relatively low nurse-patient ratios. Only for 100% cohorting rate and extremely large nurse-patient ratios, were simulations on strict cohorting network slightly lower than that on the random network.

To examine why the stochastic simulation results on the random network were similar to those on the strict cohorting network for different cohorting rates or nurse-patient ratios, we re-plotted these three subplots without considering indirect transmission via free-living bacteria, as shown in Fig. 3 (d)–(f). Figs. 3 (e) and (f) show that for a clean environment and relatively large nurse-patient ratios, the mean number of colonized patients simulated on the strict cohorting network was much lower than that on the random network. This implies that in such a scenario a strict cohorting strategy is effective in lowering the number of colonized patients. It is interesting to note that Figs. 3 (e) and (f) show that the number of colonized patients initially increases with increasing nurse-patient ratios but then declines after the ratio reaches the threshold. We also found that the threshold decreases as the cohorting rate increases.

Comparison of all the subplots of Fig. 3 yields that when the nurse-patient ratio was relatively low, the pairwise model results agree well with the stochastic simulations on both the random network and the strict cohorting network (Figs. 3). In contrast, for a relatively high nurse-patient ratio, the pairwise model overestimated the infection in a contaminated environment (shown in Fig. 3 (a) and (b)). On the other hand, in a clean environment, the pairwise model findings agree well with stochastic simulation on the strict cohorting network but underestimate the infection compared to stochastic simulation on the random network (shown in Fig. 3 (d)–(f)). As such, an interesting question arises here concerning the circumstances under which the pairwise model performs well, as indicated in a previous paper^25^.

### Effect of a contaminated environment

To examine the effect of contaminated environments, we compared the simulation results for the different indirect transmission rates. Figs. 3(c) shows that an increase in the nurse-patient ratio leads to an increased average number of colonized patients for relatively high indirect transmission rates. However, when the environment becomes extremely clean and the indirect transmission rate becomes zero, the number of colonized patients initially increases with the increasing nurse-patient ratio and then decreases after the ratio reaches a threshold, as shown in Figs. 3(f)). Moreover, this threshold is influenced by the level of environmental contamination. The lower the indirect transmission rate, the lower the threshold becomes (shown in Fig. S2(b–c) in SI). Moreover, the blue stars in Figs. 3(c) and (f) show that when nurse cohorting is less strictly implemented (i.e. a random network is used), reducing the indirect transmission rate can also lead to a decreased number of colonized patients, but this decreasing effect is not as effective as that with the strict cohorting approach. Also, in such scenarios, there is no threshold for the nurse-patient ratio such that infection starts to decrease, even if the indirect transmission rate is zero. Therefore, a contaminated environment does not contribute a lot to MRAB infection but does greatly affect the effectiveness of nurse cohorting.

**Figure 4:**
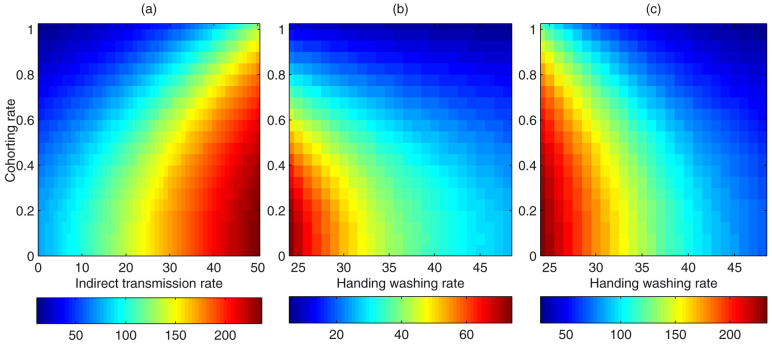
(a) The average number of colonized patients over a period of 500 days versus indirect transmission rate and cohorting rate simulated by the pairwise model. (b) ((c))The average number of colonized patients over a period of 500 days versus handwashing rate and cohorting rate when the indirect transmission rate was zero (b) or 48.9 (c) simulated by the pairwise model. All other parameter values are as listed in Table 1.

To further examine the effect of the two interventions on the number of colonized patients, we plotted heat maps of the two interventions (Fig. 4(a)) when the nurse-patient ratio was fixed at a default value (15/20). It follows from Fig. 4(a) that for a relatively large indirect transmission rate (say *v* = 49, contaminated environment), increasing the nurse cohorting rate from 0% to 50% can reduce the number of colonized patients from 234 to around 214, while reducing the indirect transmission rate from 49 to 24.5 (50%) can decrease the number of colonized patients from 234 to 151. However, for a relatively small indirect transmission rate (say, *v* = 20, relatively clean environment), either increasing the nurse cohorting rate from 0% to 50% or reducing the indirect transmission rate from 20 to 10 (50%) can result in the number of colonized patients declining similarly (from 135 to 105 or to 101). This indicates that increasing the nurse cohorting rate is not as effective as improving environmental hygiene in decreasing the number of colonized patients in the contaminated environment.

### Effect of hand washing of nurses on infection

To study the effect of increasing handwashing rates on disease control with nurse cohorting, we calculated the number of colonized patients with different rates of nurse cohorting and handwashing rates using the pairwise model. Fig. 4(b–c) show subplots of the heat map of the colonized patients versus the cohorting and handwashing rates when the indirect transmission rate was zero (b) and 48.9 (c). We varied the cohorting rate from 0% to 100% and the handwashing rate from the baseline value (*γ* = 24) to twice the baseline value (i.e., 48). Fig. 4(c) shows that when nurses are not cohorted, increasing the handwashing rate by 100% can reduce the number of colonized patients to about 71, whereas when the handwashing rate is kept at its baseline value, increasing the cohorting rate by 100% can decrease the number of colonized patients to about 110. This result indicates that when the environment is seriously contaminated (or the indirect transmission rate is relatively high), nurse cohorting is not as effective as increasing the handwashing rate. However, in clean environments (as shown in Fig. 4(b)), when nurse cohorting is not performed, increasing the handwashing rate by 100% can reduce the number of colonized patients to about 27, while when the handwashing rate is kept at its baseline value, increasing the cohorting rate by 100% can result in the number of colonized patients reducing to about 11. Hence, when the environment is clean, nurse cohorting is more effective than increasing handwashing rates in terms of reducing the number of colonized patients. This indicates that the effectiveness of these two interventions is greatly affected by the environmental contamination and consequently the implementation of these two measures should depend on the specific environmental circumstances.

## Discussion

Mathematical models play a vital role in the study of the effect of environmental contamination on bacterial transmission in hospitals^7^,^8^,^9^,^10^,^11^,^15^,^26^. Many studies^10^,^11^,^15^,^26^ implicitly assumed that the dynamics of the nurses involved are the same as those of the doctors. However, our recorded data indicated much higher contact rates between patients and nurses than between patients and doctors, which implies that the differences between doctors and nurses cannot be neglected. Therefore, distinguishing the dynamics of doctors and nurses can provide realistic descriptions, characteristic of our model, compared to existing models on hospital infections.

Environmental contamination is proven to contribute greatly to the transmission of hospital-acquired infections such as MRSA and MRAB. A number of mathematical models have been formulated by modeling the bacterial density in the environment^7^,^8^,^9^,^10^,^11^,^27^,^28^. In earlier studies, a common method was to model the environment as a black box. “Colonization” and “turnover” rates were set and parameter values were tweaked to obtain model outputs that were close to their observations^32^. Note that such observations were usually the daily data of colonized or susceptible patients and sothe earlier studies could not accurately describe the environmental contamination level, which our study was designed to do by collecting data to define the bacterial detection rate in the environment. It was represented as a proportion of positive samples, which is a sign of the degree of environmental contamination and can partially reflect the number of bacteria in the ward. Furthermore, by analyzing the correlation between the number of colonized patients and the bacterial detection rate, a power function of the daily number of colonized patients was obtained and used to describe the transmission level. It is interesting to note that this detection rate cannot only serve as a bridge between the contaminated environment and colonized patients but can also lead to a reduction of the dimension of the model. Therefore, using the data with respect to the contaminated environment, our data-driven model can capture some environmental features that the simpler model, which lacks environmental information, cannot reflect. Therefore, this data-driven model has the potential to accurately describe the contaminated environment and assess the effect of indirect transmission via free-living bacteria in the environment on infection.

Another purpose of this study was to investigate the effect of cohorting and analyze the effect of varying the number of nurses on MRAB infection. Nurse cohorting is an important measure for controlling hospital infection. Austin^15^ considered the effect of cohorting of nurses in a mean-field model by simply reducing the number of effective contacts, which ignores the heterogeneity caused by cohorting. To reflect the heterogeneity caused by cohorting, we extended the mean-field model to a pair approximation model. The proposed pairwise model treated the partnerships of individuals as the degree of nodes to reflect the heterogeneity of the network structure of the patients and cohorted nurses. Moreover, stochastic simulations on the random network and a strict cohorting network were also included in the study, which would be the benchmark, and used to compare and justify the results of the pairwise model. Also, the pairwise approximation model was needed to provide a mathematical framework to improve our understanding of heterogeneity in cohorting and indirect transmission via MRAB in the environment. Thus, our work could easily be adapted and applied to other causative pathogens of hospital-acquired infections.

In our study, different cohorting modes were investigated by varying of the cohorting rate and the nurse-patient ratio. Comparison of subfigures in Fig. 3 shows that increasing The cohorting rate is beneficial for infection control. Furthermore, in the case of a clean environment (i.e., the indirect transmission rate is zero), with strict cohorting at a high rate, we can obtain a threshold for the nurse-patient ratio; and increasing this ratio greater than the threshold may help control MRAB infection rates. This is in agreement with the results obtained by McBryde^16^ that when the HCW cohorting rate is relatively high, increasing the HCW-patient ratio to a relatively large value may lead to a decline in the number of colonized patients. However, in the case of a contaminated environment (i.e. the indirect transmission rate is relatively high), increasing the nurse-patient ratio led to an increased number of colonized patients regardless of the cohorting rate (Fig. 3 (a)–(c)). This is associated with the study of D'Agata^33^ who considered neither the contaminated environment nor cohorting, but concluded that increasing the HCW-patient ratio led to an increased number of patients harboring bacteria. These two main results imply that whether increasing nurse-patient ratios increases MRAB infection depends on the level of environmental contamination, the cohorting rate, and whether the cohorting is strictly applied.

Given a severely contaminated environment, it is more difficult to reduce the bacterial transmission rate (hence the transmission of infection) by simply increasing the nurse-patient ratio, even if nurse cohorting is strictly enforced with a 100% cohorting rate. This is because nurse cohorting can only prevent effective contacts between nurses and patients but not prevent nurses from acquiring bacteria from the contaminated environment. In such cases, increasing the number of nurses would inevitably increase the disease transmission. Therefore, it is worth noting that increasing the number of nurses only seems to be effective if there is almost 100% cohorting and the nurse-patient ratio is sufficiently large according to our Fig. 3 and Fig. S2 in the SI when the environment is relatively clean. Moreover, MRAB transmission in the ward can be fully controlled if the environment is clean, all of the HCWs are cohorted, and the HCW-patient ratio is 1:1 (not shown here), which agrees with results of McBryde^16^. Meanwhile, the threshold obtained in such a scenario is similar to that in the paper of McBryde^16^, in which neither environmental contamination nor difference between doctors and nurses was considered.

It is well known that increasing the cohorting rate and cleaning the environment more frequently are two effective interventions for controlling bacterial transmission in wards. Our results indicate that the transmission could not be fully controlled by the use of cohorting only if the environment was contaminated. This finding differs from those of earlier papers^15^,^16^ that considered cohorting only and found that a disease can be eradicated by the use of cohorting alone when all of the HCWs were cohorted and the HCW-patient ratio was 1:1. Our finding suggests that both of the measures are needed for infection prevention. Moreover, the effects of handwashing and cohorting were also affected by the degree of environmental contamination. When an environment is seriously contaminated, increasing the handwashing rate is much more effective than cohorting and should be given priority. However, when the environment is sufficiently clean, increasing the handwashing rate is not as effective as cohorting, although it can still reduce the number of colonized patients to a very low level. Therefore, in summary, cohorting and increasing the number of nurses are two effective measures that are suitable for situations in which the environment is relatively clean, while cleaning and handwashing are two effective strategies that are applicable to situations in which the environment is contaminated.

Note that the networks considered here were only between HCWs and patients and the way that environmental transmission was modeled was completely homogenous with respect to the total number of patients colonized due to a lack of reliable environmental data. However, if the nurses are cohorted then they should more often contact the environment around their assigned patients. It is interesting to argue that the effectiveness of cohorting is conditional upon the heterogeneity of the environmental contamination. Obviously, for a clean environment, not considering heterogeneity of the environmental contamination does not influence the main conclusion on effectiveness of cohorting and varying the nurse-patient ratio. In contrast, for the seriously contaminated environment, cohorting does limit nurses as then they only contact their assigned patients and their local environment, which reduces either the direct transmission rate (as our model structure described) or the indirect transmission rate or both. Then, cohorting in such a scenario may be more effective in lowering MRAB infection than our results predict. Moreover, the threshold for the nurse-patient ratio, shown in Fig. 3(f) or Fig. S2(b–c) in the SI, may appear for lower cohorting rates or dirtier environments, for which our model results predict that increasing the nurse-patient ratio consistently increases the MRAB infection. Thus, a model that explicitly assigns nurses to a certain area of the ward and models environmental contamination heterogeneously in distinct areas may produce qualitatively similar results, but under different intensities of interventions such as cohorting and varying the nurse-patient ratio.

This study aimed to explore in simulation the behavior of the proposed model which was inspired by the data. The data on detection rates, representing the degree of environmental contamination, may have much noise, which may affect our conclusion, but we hope that the approaches we used here can be applied more generally. The proposed model provides a new way to model indirect transmission via free-living bacteria in the environment and to investigate the effect of contaminated environments on MRAB infection. However, some assumptions, like the probability of colonization (per contact) between patients and doctors or nurses being the same and indirect transmission rates between HCWs and patients taken as equivalent, were made to simplify the process of parameter estimation due to lack of data. Also, the networks considered in our study are static and may not be exactly the same as the actual networks, in practice. Obtaining more realistic networks and proposing a new model structure that describes heterogeneity of environments when the cohorting measure is implemented is challenging, and we leave these topics for future studies. We examined the effects of control measures such as cleaning the environment and cohorting nurses on MRAB infection and found that their efficacy was conditional upon different environments. However, the cost effectiveness of these control measures was not considered in our study. Comprehensive control strategies from the efficacy and cost effectiveness points of view must be examined in future work.

## Author Contributions

X.W., Y.C., Y.X., L.H. and H.G. designed the study, carried out the analysis and contributed to writing the paper. X.W. performed numerical simulations. W.Z., Y.W., Q.S., H.L., J.Z., X.L.H. and X.H.H. carried out the experiment.

## Supplementary Material

Supplementary Information Supporting information

## Figures and Tables

**Table 1:** Definitions of the parameters used in the model

Parameters	Definition (Units)	Value	References
Λ	Inflow rate of patients to hospital (/day)	1.72	data
*d*_1_	Outflow rate of uninfected patients (/day)	1/4.2	data
*d*_2_	Outflow rate of infected patients (/day)	1/12.9	data
*φ*	Proportion of patients already colonized	0.0448	data
	when being hospitalized		
*β*_1_	Transmission rate from patients to doctors (/person/day)	0.078	estimated
*β*_2_	Transmission rate from patients to nurses (/person/day)	0.3588	estimated
*β*_3_	Transmission rate from doctors to patients (/person/day)	0.0061	estimated
*β*_4_	Transmission rate from nurses to patients (/person/day)	0.0281	estimated
*v*_1_	Indirect transmission rate between environments and doctors	48.9	estimated
*v*_2_	Indirect transmission rate between environments and nurses	48.9	estimated
*N_D_*	Number of doctors	11	data
*N_H_*	Number of nurses	15	data
*N_P_*	Number of beds	20	data
*γ*	Hand washing rate of HCWs (/HCW/day)	24	Ref. 15

**Table 2:** Statistical features of the estimated parameters

Parameters	mean	standard deviation	95% confidence interval	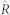
*β*_1_	0.078	0.0086	[0.072, 0.095]	1.002
*β*_3_	0.0061	0.0001	[0.006, 0.0065]	1.001
*v*	48.9	0.0410	[45.6, 58.7]	1.016
