# Supplementary material for: A data-driven mathematical model of multi-drug resistant Acinetobacter baumannii transmission in an intensive care unit
Source: Sci Rep. 2015 Mar 25;5:9478. doi: 10.1038/srep09478 (PMC4372795; doi:10.1038/srep09478)
Supplement: Supplementary Information — Supporting information [file srep09478-s1.pdf]

A data-driven mathematical model of multi-drug resistant Acinetobacter baumannii transmission in an intensive care unit

Xia Wang, Yong Chen, Wei Zhao, Yan Wang, Qing Song, Hui Liu, Jingya Zhao, Xuelin Han, Xiaohua Hu, Hajo Grundmann, Yanni Xiao[1], Li Han[1]

[1]Corresponding author. E-mail: hanlicdc@163.com; yxiao@mail.xjtu.edu.cn

In this supplementary material, we provide the descriptions of The contact rate, the detailed deduction for the relationship between the detection rate and the number of colonized patients, the pair-approximation model with cohorting of nurses, the detailed configuration of two kinds of networks and the event-driven stochastic simulation method on the network.

1 The detailed description of contact rate

”Contact” was defined as any physical contact with a patient or the patient’s surroundings or indwelling devices as described previously[1] . The contact rate was defined as the number of contacts per patient per hour. The contact rates between patients and nurses or doctors were obtained through direct observation for a total of 59 hours over 37 days in August, September, and October 2008. Observations were mainly conducted in the morning and afternoon shifts (about 8 hours during the daytime). All of the patients present during the investigation time were observed. The average duration of each observation per patient was 6.0 ± 1.7 minutes. The total numbers of contacts during the observation periods were 1,236 for nurses and 267 for doctors. Our data show that the contact rates in the different periods might be very different, which made it difficult to obtain an accurate contact rate per unit of time. However, the contact rates for the morning and afternoon shifts were 5.8 and 1.1 for the doctors and 24.2 and 12.1 for the nurses, respectively. So, we could deduce that the contact rate of patients with nurses was approximately 4.6 times greater than that of patients with doctors.

2 The relationship between the detection rate and number of colonized patients

The detection rate of MRAB in the environment is defined by the proportion of positive samples, which could represent the concentration of bacteria in the environment. As we know, the bacteria in the environment come from colonized patients. So, there may be a certain correlation between the detection rate and the number of colonized patients. Comparing Figs.1(a) and 1(b) in the main text suggests that these two data sets have similar trends. Moreover, Pearson’s correlation coefficient was calculated to be 0.57

($p = 7.24 * 10^{-5}$) between the number of colonized patients and the detection rates of bacteria in a ward. Further, to clearly and more directly show the relationship between the number of colonized patients and the MRAB detection rates, we used a linear function, a exponential function and a power-law function to fit the mean detection rates. We calculated the R^2 coefficient of determination which is a statistical measure of how well the regression line approximates the real data points. We foundd that the R^2 of the power law function was the biggest (i.e., 0.8878) among the three R^2 coefficients. Hence, a power-law dependence was chosen to fit the data. Let $W(t)$ be the detection rate of MRAB in the ICU environment and $P^C(t)$ be the number of colonized patients, then we have $W(t) = a(P^C(t))^b, a = 0.0032, b = 1.54$, and the goodness of fit was shown in Fig.1(c) of the main text. In the perspective of biological meaning, this relationship is sensible.

3 The pair-approximation model with cohorting of nurses

Here we provide more detailed descriptions of the pair-approximation model with cohorting of nurses. The parameter p is defined to be the proportion of nurses who are cohorted. With the cohorting of nurses, the total number of nurses N_H is divided into $N_H^1 = pN_H$ and $N_H^2 = (1 - p)N_H$. N_H^1 represents the total number of nurses who are cohorted and N_H^2 represents the number of nurses who are not cohorted. H_1^C and H_2^C represent the number of colonized nurses who are cohorted and not cohorted, respectively. Cohorted nurses were considered to be maximum grouped. So, the degree of cohorted nurses Q_H^1 is $\frac{N_P}{N_H^1}$ and the degree of the other nurses is $Q_H^2 = N_P$. Therefore, the degree of patients is $Q_P = (Q_D N_D + Q_H^1 N_H^1 + Q_H^2 N_H^2)/N_P$. The model is as follows:

$$
\begin{cases}
\frac{d[D^C]}{dt} = \tau_1[P^C D^S] + \nu_1(N_D - [D^C])W - \gamma[D^C] \\
\frac{d[H_1^C]}{dt} = \tau_2[P^C H^S] + \nu_2(N_H^1 - [H_1^C])W - \gamma[H_1^C] \\
\frac{d[H_2^C]}{dt} = \tau_5[P^C H_2^S] + \nu_2(N_H^2 - [H_2^C])W - \gamma[H_2^C] \\
\frac{d[P^S]}{dt} = (1-\varphi)\widehat{\Lambda}[\phi] - \tau_3[D^C P^S] - \tau_4[H_1^C P^S] - \tau_6[H_2^C P^S] - d_1[P^S] \\
\frac{d[P^C]}{dt} = \varphi\widehat{\Lambda}[\phi] + \tau_3[D^C P^S] + \tau_4[H_1^C P^S] + \tau_6[H_2^C P^S] - d_2[P^C] \\
\frac{d[D^C P^C]}{dt} = \tau_3([D^C P^S D^C] + [D^C P^S]) + \tau_4[D^C P^S H_1^C] + \tau_6[D^C P^S H_2^C] - \gamma[D^C P^C] \\
\qquad +\varphi\widehat{\Lambda}[D^C\phi] - d_2[D^C P^C] + \tau_1([P^C D^S P^C] + [D^S P^C]) + \nu_1[D^S P^C]W \\
\frac{d[H_1^C P^C]}{dt} = \tau_4([H_1^C P^S H_1^C] + [H_1^C P^S]) + \tau_3[H_1^C P^S D^C] + \tau_6[H_1^C P^S H_2^C] - \gamma[H_1^C P^C] \\
\qquad +\varphi\widehat{\Lambda}[H_1^C\phi] - d_2[H_1^C P^C] + \tau_2([P^C H^S P^C] + [H^S P^C]) + \nu_2[H^S P^C]W \\
\frac{d[H_2^C P^C]}{dt} = \tau_6([H_2^C P^S H_2^C] + [H_2^C P^S]) + \tau_3[H_2^C P^S D^C] + \tau_4[H_2^C P^S H_1^C] - \gamma[H_2^C P^C] \\
\qquad +\varphi\widehat{\Lambda}[H_2^C\phi] - d_2[H_2^C P^C] + \tau_5([P^C H_2^S P^C] + [H_2^S P^C]) + \nu_2[H_2^S P^C]W
\end{cases}
\tag{1}
$$

Similar to model (2), but only a part of the nodes and pairs is included in the equation and others can be derived from the following equalities.

$$
\begin{aligned}
&[D^C P^S] + [D^C P^C] + [D^C\phi] = Q_D[D^C], \\
&[D^S P^S] + [D^S P^C] + [D^S\phi] = Q_D[D^S], \\
&[H_i^C P^S] + [H_i^C P^C] + [H_i^C\phi] = Q_H^i[H_i^C], \\
&[H_i^S P^S] + [H_i^S P^C] + [H_i^S\phi] = Q_H^i[H_i^S], \quad i = 1, 2, \\
&[D^C P^C] + [D^S P^C] = Q_{P_1}[P^C], \ \ [H_1^C P^C] + [H_1^S P^C] = Q_{P_2}[P^C], \\
&[H_2^C P^C] + [H_2^S P^C] = Q_{P_3}[P^C], \ \ [D^C P^S] + [D^S P^S] = Q_{P_1}[P^S], \\
&[H_1^C P^S] + [H_1^S P^S] = Q_{P_2}[P^S], \ \ [H_2^C P^S] + [H_2^S P^S] = Q_{P_3}[P^S], \\
&[D^C\phi] + [D^S\phi] = Q_{P_1}[\phi], \ \ [H_1^C\phi] + [H_1^S\phi] = Q_{P_2}[\phi], \\
&[H_2^C\phi] + [H_2^S\phi] = Q_{P_3}[\phi], \ \ [D^S] + [D^C] = N_D, \\
&[H_1^S] + [H_1^C] = N_H^1, \ \ [H_2^S] + [H_2^C] = N_H^2, \\
&[P^S] + [P^C] + [\phi] = N_P, \ \ Q_{P_1}N_P = Q_D N_D, \\
&Q_{P_2}N_P = Q_H^1 N_H^1, \ \ Q_{P_3}N_P = Q_H^2 N_H^2.
\end{aligned}
$$

Values of parameters corresponding to node events in the pairwise model were the same as those in the mean-field model (1) except for the admission rate. The admission of patients in the mean-field model is a constant input but depends on the number of beds that are available, so we assume that it is proportional to the number of empty nodes in the contact network. Hence, in the pairwise model (2), the admission rate $\widehat{\Lambda}$ is assumed

to be $\frac{\Lambda}{E}$, where E is the average number of empty beds in the ward obtained from our data. According to the result in the paper of Keeling[2] , the rates of edge events in the pairwise model can be converted from the rates for the mean-field model according to the following equalities:

$$\tau_1 = \beta_1 \frac{N_P}{Q_D}, \quad \tau_2 = \beta_2 \frac{N_P}{Q_H}, \quad \tau_3 = \beta_3 \frac{N_D}{Q_{P_1}}, \quad \tau_4 = \beta_4 \frac{N_H}{Q_{P_2}}.$$

4 Configuration of two networks

Two kinds of networks are considered in this paper. Specifically, for the strict cohorting network, edges are connected strictly according to the cohort so that the degree of every node is fixed. While, for the random network,[3] it is not the real degrees but the average degrees for doctors and nurses that are the same as those of the strict cohorting network because edges are connected randomly. Therefore, the random network is generated by random connection of two nodes when average degrees of all kinds of nodes are given. The strict cohorting network is a special random network in which the edges are strictly connected according to the grouping.

5 Stochastic simulation on the network

Event-driven stochastic simulation[4] on the network was conducted in this paper. According to the Gillespie algorithm,[5] the key points of the event-driven stochastic simulation are how to determine the time to next event and which event happens every time. Sum all of the rates of possible events together, denoted by R_{total} - the total event rates, and view the event process as a Poisson process, then the time interval between every two events follows an exponential distribution. So, the time until the next event can be obtained by sampling a random number r_1 from the exponential distribution with parameter R_{total}. To determine the next event in a stochastic simulation, the rates of all possible events should be ordered in an array first. Then, the cumulative sum of the array can be obtained, which can be used to choose the next event by sampling a random number r_2 from a uniform distribution within $[0, R_{total}]$ and choosing the first event, such that r_2 is less than the rate associated with that event. It is worth noting that stochastic simulation on a contact net-

work, each event impinges on the state of only its neighborhood of contacts. So, if an event is chosen to occur to a node, both the event rate of this node and its neighbors should be updated accordingly.

The main steps of the algorithm can be summarized as follows:

1. Set the time value $t = 0$ and initialize all the states and event rates of every nodes in a given network.

2. Generate random numbers r_1 and r_2 to determine the time to next event and which the next event may occur.

3. Increase the time step by the randomly generated time r_1 and update states of the associated nodes.

4. Go back to Step 2 unless the number of reactants is zero or the simulation time has been exceeded.

References

1. Grundmann H, Hori S, Winter B, Tami A & Austin D.J. Risk factors for the transmission of methicillin-resistant Staphylococcus aureus in an adult intensive care unit: fitting a model to the data. *J. Infect. Dis.* **185**, 481-488 (2002).

2. Keeling, M. J. The effects of local spatial structure on epidemiological invasions. J. R. Soc. Interface. **2**, 295-307 (1999).

3. Keeling, M. J. & Eames, K. T. D. Networks and epidemic models. *Proc. R. Soc. Lond. B.* **266**, 859-867 (2005).

4. Keeling, M. J. & Rohani, P. *Modeling Infectious Diseases In Humans And Animals,* Princeton University Press, USA (2008).

5. Gillespie, D. T. & Eames, K. T. D. Exact Stochastic Simulation of Coupled Chemical Reactions. *J. Phys. Chem.* **81**, 2340-2361 (1997).

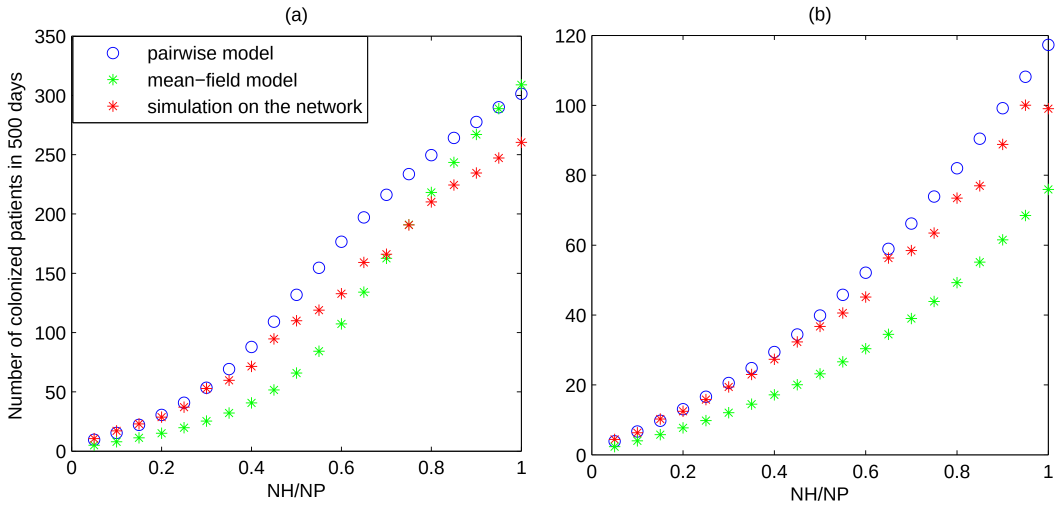

Figure S1: The predicted average number of colonized patients in a period of 500 days versus the ratio of nurses to patients from the mean-field model (green stars), the pairwise model (blue circles) and the stochastic simulation (red stars) on the random network when $Q_H = NP$. (a) The indirect transmission rate is 48.9. (b) The indirect transmission rate is 0. All other parameters are as listed in Table 1.

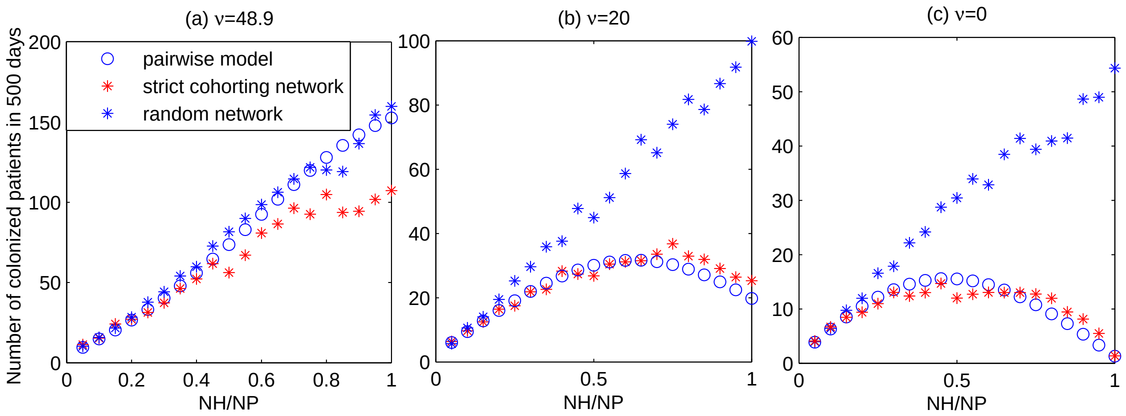

Figure S2: (a)-(c) The predicted average number of colonized patients in a period of 500 days versus the nurse-patient ratio when the cohorting rate was 100% and the indirect transmission rate was 48.9 (a), 20 (b), or 0 (c). Blue circles show simulation results for the pairwise model. Blue (red) stars show simulation results on the random network (the strict cohorting network). All other parameters are as listed in Table 1.
